# Human toxoplasmosis: a systematic review for genetic diversity of *Toxoplasma gondii* in clinical samples

**DOI:** 10.1017/S0950268818002947

**Published:** 2018-11-05

**Authors:** S. A. Hosseini, A. Amouei, M. Sharif, Sh. Sarvi, L. Galal, J. Javidnia, A. S. Pagheh, S. Gholami, A. Mizani, A. Daryani

**Affiliations:** 1 Toxoplasmosis Research Center, Mazandaran University of Medical Sciences, Mazandaran, Sari, Iran; 2 Student Research Committee, Mazandaran University of Medical Sciences, Mazandaran, Sari, Iran; 3 Department of Parasitology and Mycology, School of Medicine, Mazandaran University of Medical Science, Mazandaran, Sari, Iran; 4 Department of Parasitology School of Medicine, Sari Branch, Islamic AZAD University, Sari, Iran; 5 Neuroépidémiologie Tropicale, Laboratoire deParasitologie-Mycologie, Facultéde Médecine, Université deLimoges, Limoges 87025, France

**Keywords:** AIDS, congenital, genotype, ocular, *Toxoplasma gondii*

## Abstract

*Toxoplasma gondii* (*T. gondii*) as an obligate intracellular protozoan with a worldwide distribution can infect virtually all warm-blooded animals and humans. This study aims to provide a summary of the available data on genotypes of *T. gondii* in human. Five databases including MEDLINE in PubMed, Scopus, Science Direct, Web of Science and Google Scholar were searched for the *T. gondii* genotyping in human during 1995–August 2017. Next, we screened all the articles based on the inclusion and exclusion criteria. Overall, 26 studies were eligible regarding genotyping *T. gondii* in human samples. In clonal genotyping, 167 out of 286 cases (58%) were infected with type II. Genetic characterisation of *T. gondii* isolates displayed that type II was the most predominant genotype in human with the prevalence of 64.3%, 62.1% and 41.7% in patients with AIDS, congenital and ocular toxoplasmosis, respectively. In ToxoDB genotyping, most individuals were infected with genotypes #9 and #65 (21.2%). Based on these results, genotype profile of *T. gondii* isolates is different throughout the world. The strains in Asian and African countries are characterised by low genetic diversity, while in North and South America a wide diversity of this parasite is found. In countries without any data (e.g. Australia, Western and Southern Africa and Western Asia), identification of *T. gondii* genotypes might discover higher genetic diversity.

## Introduction

*Toxoplasma gondii* as an obligate intracellular protozoan with a worldwide distribution can infect virtually all warm-blooded animals and human. One-third of the human population is assumed to be infected with *T. gondii* [1]. In immunocompetent individuals, clinical symptoms of toxoplasmosis present as mild and self-limiting, including fever, malaise and lymphadenopathy. However, infection is usually more severe in immunocompromised people and pregnant women. In these groups, the infection is accompanied by more complications, such as encephalitis, retinochoroiditis, foetus abortion, splenomegaly and pneumonitis [2].

The number and genetic diversity of parasites play an important role in pathogenesis of *T. gondii* [3]. Identifying the genotypes of *T. gondii* using molecular technologies is invaluable in epidemiological studies. These techniques include multilocus polymerase chain reaction-restriction fragment length polymorphism (PCR-RFLP), microsatellite analysis, random amplified polymorphic (RAPD), multilocus sequence typing (MLST) and high-resolution melting (HRM) analysis [4].

The first genotyping studies on *T. gondii* strains in France and the United States of America demonstrated a clonal population structure with three main lineages namely type I, II and III [5]. Despite the sexual phase and global distribution of *T. gondii*, the population structure of this parasite is explained as highly clonal with low genetic diversity [6]. This was found in genetic researches on isolates from Europe and USA which classified the isolates as three major genotypes of type I, II and III (approximately 90%) [7].

More genetic variation of *T. gondii*, except for the three main lineages, was found in the other continents. These ‘new’ genotypes were designated as atypical, exotic, recombinant or non-archetypal genotypes [8, 9]. Multilocus PCR-RFLP analysis of 10 gene markers in 1457 *T. gondii* isolates worldwide revealed 189 different genotypes. The data of these genotypes are displayed in ToxoDB [10].

Numerous studies were performed on *T. gondii* genotyping in different groups of patients and healthy individuals. However, there is no exhaustive documented data regarding this subject. Therefore, this study aims to provide a summary of the available data on genotypes of *T. gondii* in human. This study was carried out to evaluate the predominant genotypes in different continents of the world. Moreover, the prevalence of genotypes is assessed in various groups of human toxoplasmosis, namely immunocompromised, congenital, ocular toxoplasmosis, cancer and immunocompetent.

## Methods

This review followed the preferred reporting items for systematic reviews (PRISMA) guidelines [11].

### Literature search, study selection and data extraction

We searched MEDLINE in PubMed, Scopus, Science Direct, Web of Science and Google Scholar during 1995–August 2017. The utilised keywords entailed *Toxoplasma gondii*, toxoplasmosis, genotype, genotyping, molecular characterisation, human, individuals, patients, pregnancy, women, congenital, cancer, AIDS, HIV, immunocompromised and ocular toxoplasmosis. We searched the mentioned keywords alone and in combination with the others.

In this systematic review, the exclusion criteria encompassed not describing the *T. gondii* genotypes in human, in addition to using genetic characterisation methods with less than five gene markers. To collect precise information, a comprehensive search was completed on all published and unpublished articles including full texts, abstracts and parasitology congresses summaries. All the data were collected from English language articles. We defined a protocol for data extraction and two authors assessed the obtained data independently. Afterwards the disagreements between the comments were discussed and resolved.

The data extracted from studies encompassed year, first author, country and continent, total sample size, methods, genotypes of *T. gondii*, type of patients and molecular markers used for genotyping in the study.

The genotypes were classified as classical types I, II, III, mix/recombinant (i.e. I&III, I&II, II&III/exotic and unknown) atypical, Africa I and ToxoDB genotype. A protocol of this systematic review with CRD 42017070501 code is available in international prospective register of systematic reviews (PROSPERO, http://www.crd.york.ac.uk/prospero) [12].

## Results

**Fig. 1.**
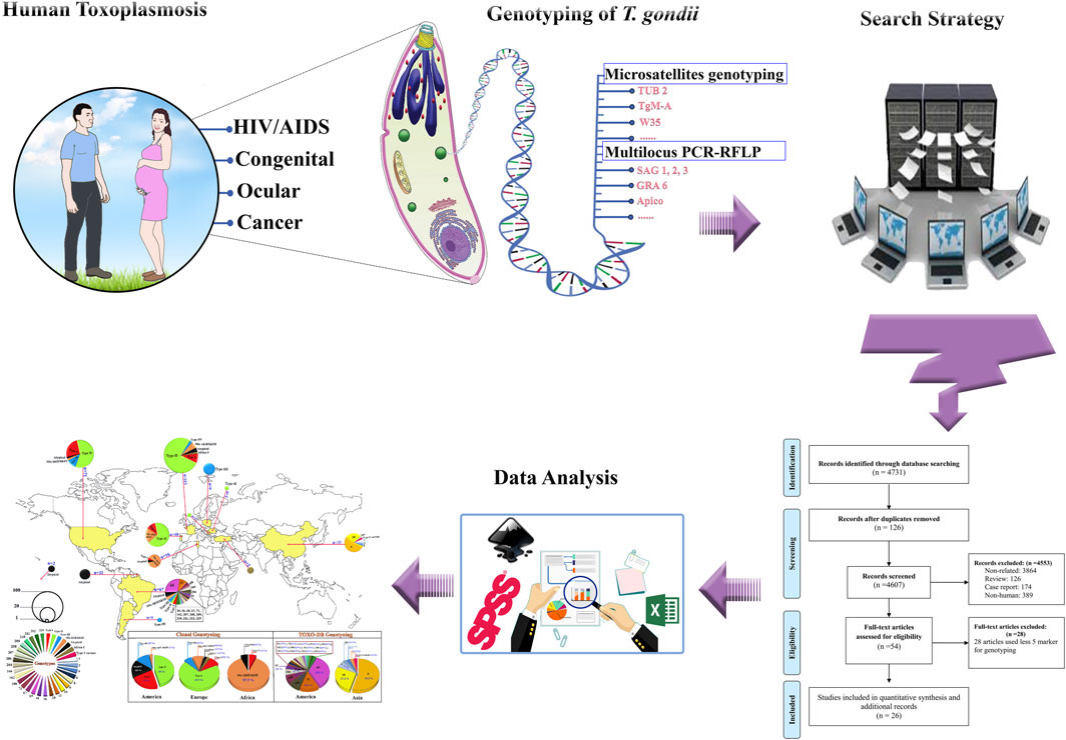
Graphic summary of the study on genetic diversity of *T. gondii* in human.

**Fig. 2.**
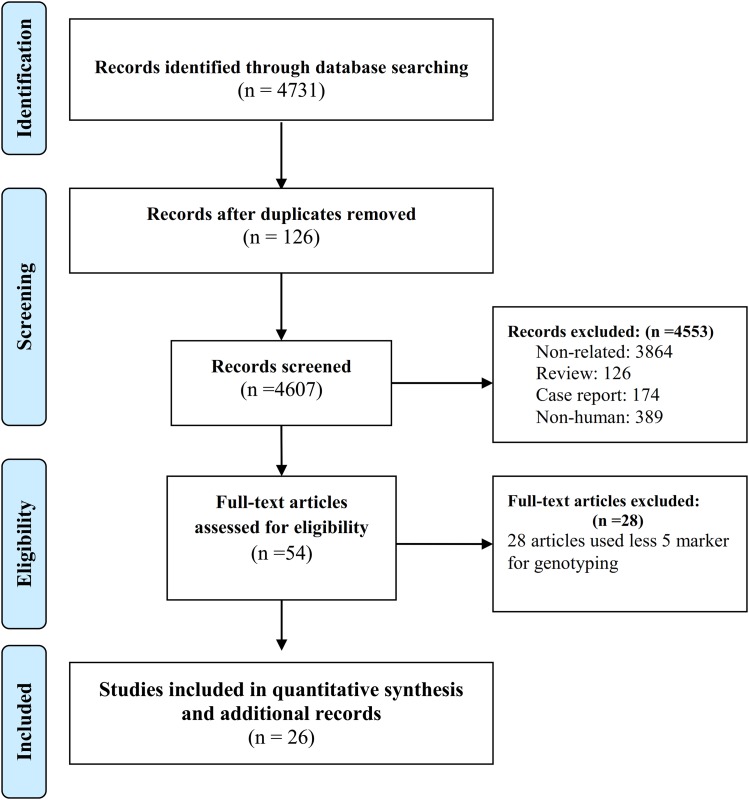
PRISMA flow diagram describing the study design.

A graphic summary of this study on genetic diversity of *T. gondii* is shown in Figure 1. Overall, we selected 4731 studies for this systematic review. After screening, 26 papers were eligible as genotyping studies on *T. gondii* in human samples (Table 1 and Fig. 2). From these papers, we collected 371 individual genotyping data, 286 of which being about clonal genotyping (I, II, III, mix/recombinant, Africa I and atypical) and 85 about ToxoDB genotyping (genotypes #1–#231).

**Table 1.** Overview of the included studies regarding genotyping *T. gondii* isolates from human and summary of the main findings

No.	Author	Patients group	Methods for genotyping	Molecular markers for genotyping	No. of isolates	No. (%) genotype
1	Howe and Sibley [13]	AIDS/congenital toxoplasmosis	PCR-RFLP	SAG1, SAG2, L328, ROP1, 850, 62	68	16 (23.5) I, 43 (63.2) II, 9 (13.3) III
2	Grigg *et al*. [9]	Ocular toxoplasmosis	PCR-RFLP	B1, SAG1, SAG2, SAG3, SAG4	12	3 (25) I, 3 (25) II, 1 (8.4) III, 5 (41.6) I&III
3	Ajzenberg *et al*. [14]	Congenital toxoplasmosis	PCR of MS markers	TUB2, W35, TG-MA, w35487, N60608, N82375, N83021, N61191, AA519150	86	73 (84.8) II, 7 (8.1) I, 2 (2.3) III, 4 (4.6) atypical
4	De melo Ferreira [15]	Congenital toxoplasmosis	PCR-RFLP	SAG1, SAG2, SAG3, B1, cB21-4, cS10-A6, GRA6, L363	4	4 (100) atypical
5	Khan *et al*. [16]	Ocular toxoplasmosis	PCR-RFLP	5′SAG2, 3′SAG2, BTUB, GRA6, SAG3	11	3 (27.3) I, 2 (18.1) II, 3 (27.3) III, 3 (27.3) I&III
6	Nowakowske *et al*. [17]	Newborns	PCR-RFLP	3′SAG2, 5′SAG2, SAG3, BTUB, GRA6	9	9 (100) III
7	Djurković-Djaković *et al*. [18]	Congenital toxoplasmosis	PCR-RFLP	5′SAG2, 3′SAG2, BTUB, SAG3, GRA6	1	1 (100) II
8	Genot *et al*. [19]	AIDS	PCR of MS markers	TUB2, TgM-A, W35, B17, B18	1	1 (100) I&III
9	Demar *et al*. [20]	Congenital immunocompetent	PCR of MS markers	TUB2, W35, TgM-A, B18, B17	11	11 (100) atypical
10	Zhou *et al*. [21]	ND	Mn-PCR-RFLP	SAG1, SAG2, SAG3, BTUB, GRA6, c22-8, c29-2, L358, PK1, Apico	3	1 (33.3) #1, 1 (33.3) #3, 1 (33.3) #4
11	Boughattes *et al*. [22]	Congenital toxoplasmosis	PCR-RFLP	3′SAG2, 5′SAG2, SAG3, BTUB, GRA6, APICO	14	1 (7.14) I, 7 (50) I&III, 3 (21.4) I&II, 3 (21.4) I&II + I&III
12	Ferreira *et al*. [23]	AIDS, congenital, pregnant, ocular, acute toxoplasmosis	Mn-PCR-RFLP	SAG1, SAG2, SAG3, BTUB, GRA6, C22-2, C29-2, L358, PK1, APICO	20	18 (90) #65, 1 (5) #71, 1 (5) #6
13	Boughattes *et al*. [24]	Congenital toxoplasmosis	PCR-RFLP	3′SAG2, 5′SAG2, SAG3, AK69, APICO, UPRT1	1	1 (100) I&III
14	Boughattes *et al*. [25]	Pregnant and diabetic	PCR-RFLP	3′SAG2, 5′SAG2, SAG2, SAG3, GRA6, BTUB, APICO, PK1, KT850, UPRT1	1	1 (100) atypical
15	Fekkar *et al*. [26]	Ocular toxoplasmosis	PCR of MS markers	TUB2, W35, TG-MA, B18, B17	13	10 (76.9) II, 1 (7.7) II&III, 2 (15.4) Africa I
16	Carneiro *et al*. [27]	Newborns	Mn-PCR-RFLP	3′SAG2, 5′SAG2, SAG1, SAG3, SAG2, GRA6, L356, APICO, C29-2, C22-9, PK1	25	7 (28) #11, 1 (4) #8, 3 (12) #206, 2 (8) #41, 2 (8) #108, 1 (4) #67, 1 (4) #207, 1 (4) #162, 1 (4) #208, 1 (4) #36, 1 (4) #209, 1 (4) #210, 1 (4) #211, 1 (4) #212, 1 (4) (mix/recombinant)
18	Wang *et al*. [28]	AIDS/cancer	Mn-PCR-RFLP	SAG1, SAG2, SAG3, BTUB, GRA6, C22-2, C29-2, L358, PK1, APICO	4	1 (25) #1, 1 (25) #9, 1 (25) #10, 1 (25) #204
19	Costache *et al*. [29]	Congenital toxoplasmosis	PCR of MS markers	TUB2, W35, TG-MA, w35487, N60608, N82375, N83021, N61191, AA519150	1	1 (100) II
20	Döşkaya *et al*. [30]	Congenital toxoplasmosis	PCR of MS markers	TUB-2, W35, TgM-A, B18, B17, M33, IV.1, XI.1, M48, M102, N60, N82, AA, N61, N83	2	2 (100) Africa I
21	Pardini *et al*. [31]	Congenital toxoplasmosis	PCR-RFLP	SAG2, SAG3, BTUB, GRA6, c29-2, c22-8, L358, PK1, Apico	1	1 (100) III
22	Higa *et al*. [32]	Congenital toxoplasmosis	Mn-PCR-RFLP	SAG1, SAG2, SAG3, BTUB, GRA6, C22-2, C29-2, L358, PK1, APICO	3	3 (100) #166
23	Silva *et al*. [33]	Congenital toxoplasmosis	Mn-PCR-RFLP	SAG1, 5′SAG2, 3′SAG2, SAG2 alt, SAG3, BTUB, GRA6, c22-8, c29-2, L358, PK1, Apico	4	2 (50) #108, 1 (25) # 206, 1 (25) # 229
24	Yeraa *et al*. [34]	Congenital toxoplasmosis	PCR of MS markers	TUB2, W35, TgM-A, B18, B17, M33, IV.1, XI.1, M48, M102, N60, N82, AA, N61, N83	2	2 (100) atypical
25	Wang *et al*. [35]	Cancer	Mn- PCR-RFLP	SAG1, SAG2, SAG3, BTUB, GRA6, C22-2, C29-2, L358, PK1, APICO	9	9 (100) #9
26	Cong *et al*. [36]	Cancer	Mn-PCR-RFLP	SAG1, SAG2, SAG3, BTUB, GRA6, C22-2, C29-2, L358, PK1, APICO	17	8 (47.1) #10, 8 (47.1) #9, 1 (5.8) (type I variant)
27	Virales *et al*. [37]	Congenital toxoplasmosis	PCR-RFLP/PCR of MS markers	SAG2, TUB, TgM, W35, B17, B18	48	6 (12.5) I, 32 (66.6) II, 6 (12.5) I&II, 4 (8.3) I&III

MS, microsatellite Marker; ND, not determined; Mn, multi locus nested-PCR-RFLP (10 markers).

**Fig. 3.**
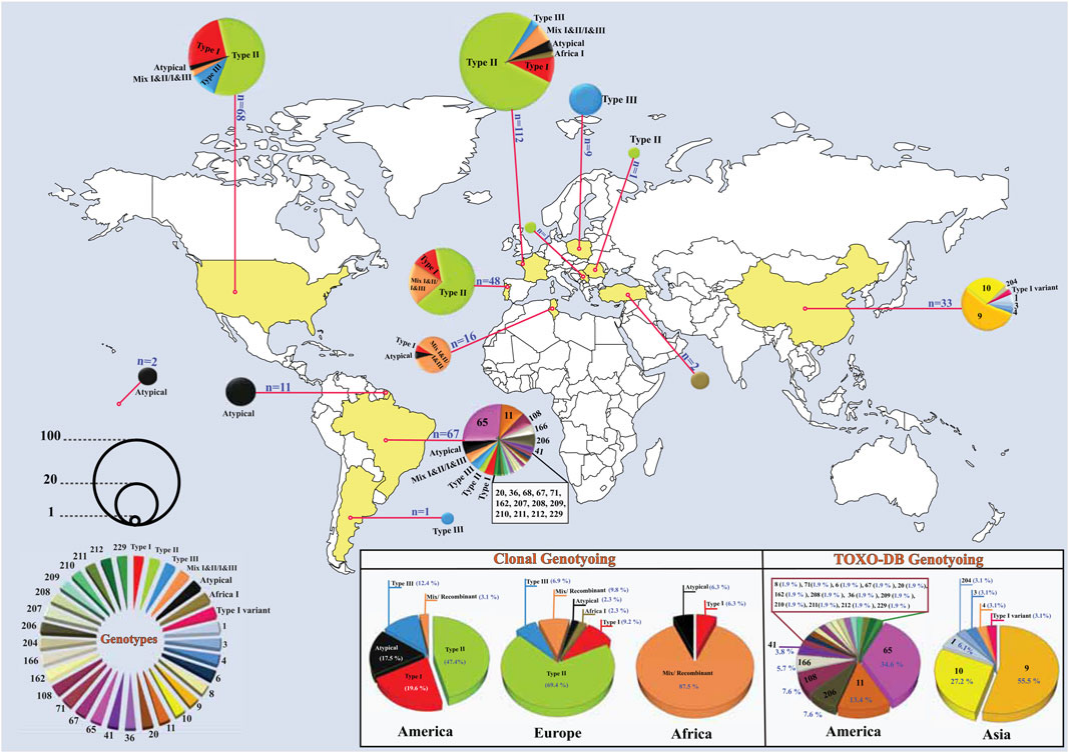
Geographical distribution of *T. gondii* strains in the world; sizes of pie charts correlate with the total number of isolates for each country; colours indicate different genotypes.

Out of 286 cases, in clonal genotyping using PCR-RFLP based on multilocus typing (i.e. more than five gene markers) and microsatellite markers, 166 (58%), 36 (12.6%), 34 (11.9%), 24 (8.4%), 22 (7.7%) and 4 (1.4%) were infected with types II, I, mix/recombinant, III, atypical and Africa I type, respectively (Table 2). The global prevalence rate of *T. gondii* clonal genotypes in different continents is indicated in Figure 3. The most prevalent types were I and III reported from America (19/97, 19.6% and 12/97, 12.4%, respectively) and type II from Europe (120/173, 69.4%) (Table 3).

**Table 2.** Clonal genotyping of *T. gondii* based on multilocus PCR-RFLP and microsatellite in different groups

Group	Author	Country	No. of isolates	Type I No. (%)	Type II No. (%)	Type III No. (%)	Mix/recombinant, Africa I and atypical No. (%)
Congenital	Howe and Sibley [13]	USA	41	10 (24.4)	25 (61)	6 (14.6)	
	Ajzenberg *et al*. [14]	France	86	7 (8.1)	73 (84.9)	2 (2.3)	4 (4.6) atypical
	Ferreira *et al*. [23]	Brazil	4				4 (100) atypical
	Nowakowske *et al*. [17]	Poland	9			9 (100)	
	Djurković-Djaković *et al*. [18]	Serbia	1		1 (100)		
	Demar *et al*. [20]	French Guiana	3				3 (100) atypical
	Boughattes *et al*. [22]	Tunisia	14	1 (7.1)			13 (50) mix/recombinant
	Boughattes *et al*. [24]	Tunisia	1				1 (100) mix/recombinant
	Boughattes *et al*. [24]	Tunisia	1				1 (100) atypical
	Costache *et al*. [29]	Romania	1		1 (100)		
	Pardini *et al*. [31]	Argentina	1		1 (100)		
	Döşkaya *et al*. [30]	Turkey	2				2 (100) Africa I
	Yera *et al*. [34]	French Polynesia	2				2 (100) atypical
	Vilares *et al*. [37]	Portugal	48	6 (12.5)	32 (66.7)		10 (20.8) mix/recombinant
	Total		214	24 (11.2)	133 (62.1)	17 (7.9)	24 (11.2) mix/recombinant, 14 (6.5) atypical, 2 (0.9) Africa I
AIDS	Howe and Sibley [13]	USA	27	6 (20)	18 (70)	3 (10)	
	Genot *et al*. [19]	France	1				1 (100) mix/recombinant
	Total		28	6 (21.4)	18 (64.3)	3 (10.7)	1 (3.6) mix/recombinant
Ocular	Grigg *et al*. [9]	France	12	3 (25)	3 (25)	1 (8.4)	5 (41.7) mix/recombinant
	Khan *et al*. [16]	Brazil	11	3 (27.2)	2 (18.2)	3 (27.2)	3 (27.2) mix/recombinant
	Fekkar *et al*. [26]	France	13		10 (77)		1 (7.7) mix/recombinant, 2 (15.4) Africa I
	Total		36	6 (16.7)	15 (41.7)	4 (11.1)	9 (22.2) mix/recombinant, 2 (5.6) Africa I
Immunocompetent	Demar *et al*. [20]	French Guiana	8				8 (100) atypical
Total			286	36 (12.6)	166 (58)	24 (8.4%)	34 (11.9) mix/recombinant, 22 (7.7) atypical, 4 (1.4) Africa I

**Table 3.** Global prevalence rate of clonal genotypes of *T. gondii* based on microsatellite and multilocus PCR-RFLP by continent

Continent	No. of isolates	Type I No. (%)	Type II No. (%)	Type III No. (%)	Mix/recombinant No. (%)	Atypical No. (%)	Africa I No. (%)
America	97	19 (19.6)	46 (47.4)	12 (12.4)	3 (3.1)	17 (17.5)	–
Africa	16	1 (6.3)	–	–	14 (87.5)	1 (6.3)	–
Europe	173	16 (9.2)	120 (69.4)	12 (6.9)	17 (9.8)	4 (2.3)	4 (2.3)
Total	286	36 (12.6)	166 (58)	24 (8.4)	34 (11.9)	22 (7.7)	4 (1.4)

In ToxoDB genotyping using PCR-RFLP based on multilocus typing, most individuals were infected with genotypes #9 and #65 (21.2%). It should be noted that genotype #65 was isolated from America, and genotype #9 from Asia (Table 4 and Fig. 3).

**Table 4.** Genetic characterisation of *T. gondii* based on multilocus PCR-RFLP (ToxoDB) in different groups

Group	Author	Country	Type of sample	No. of isolates	No. (%) ToxoDB genotype
AIDS	Ferreira *et al*. [23]	Brazil	CSF	9	7 (#65), 1(#71), 1 (#6)
	Wang *et al*. [28]	China	Blood	2	1 (#9), 1 (#10)
	Total			11	7 (63.6) #65, 1 (9.1) #71, 1 (9.1) #6, 1 (9.1) #9, 1 (9.1) #10
Congenital	Ferreira *et al*. [23]	Brazil	Amniotic fluid	1	1 (100) #65
	Ferreira *et al*. [23]	Brazil	Blood	1	1 (100) #65
	Carneiro *et al*. [27]	Brazil	Blood	25	7 (28) #11, 1 (4) #8, 3 (12) #206, 2 (8) #41, 2 (8) #108, 1 (4) #67, 1 (4) #207, 1 (4) #162, 1 (4) #208, 1 (4) #36, 1 (4) #209, 1 (4) #210, 1 (4) #211, 1 (4) #212, 1 mix/recombinant
	Silva *et al*. [33]	Brazil	Blood	4	2 (50) #108, 1 (25) # 206, 1 (25) #229
	Higa *et al*. [32]	Brazil	Blood	3	3 (100) #166
	Total			34	7 (20.5) #11, 4 (11.7) #108, 4 (11.7) #206, 3 (8.8) #166, 2 (5.8) #65, 2 (5.8) #41, 1 (2.9) # 229, 1 (2.9) #8, 1 (2.9) #67, 1 (2.9) #207, 1 (2.9) #162, 1 (2.9) #208, 1 (2.9) #36, 1 (2.9) #209, 1 (2.9) #210 1 (2.9) #211, 1 (2.9) #212, 1 (2.9) mix/recombinant
Ocular	Ferreira *et al*. [23]	Brazil	Blood	7	7 (100) #65
Cancer	Wang *et al*. [28]	China	Blood	2	1 (50) #204, 1 (50) #1
	Cong *et al*. [36]	China	Blood	17	8 (47.1) #9, 8 (47.1) #10, 1 (5.8) type I variant
	Wang *et al*. [35]	China	Blood	9	9 (100) #9
	Total			28	17 (60.7) #9, 8 (28.5) #10, 1 (3.7) #204, 1 (3.7) #1, 1 (3.7) type I variant
Unknown	Zhou *et al*. [21]	China	ND	3	1 (33.3) #1, 1 (33.3) #3, 1 (33.3) #4
Acute toxoplasmosis	Ferreira *et al*. [23]	Brazil	Blood/CSF	2	2 (100) #65
Total				85	18 (21.1) # 9, 18 (21.1) #65, 9 (10.5) #10, 7 (8.2) #11, 4 (4.7) #206, 4 (4.7) #108, 3 (3.5) #166, 2 (2.3) #41, 3 (2.3) #1, 2 (2.3) #204, 1 (1.1) #8, 1 (1.1) #71, 1 (1.1) #6, 1 (1.1) #67, 1 (1.1) #20, 1 (1.1) #162, 1 (1.1) #208, 1 (1.1) #36, 1 (1.1) #209, 1 (1.1) #210, 1 (1.1) #211, 1 (1.1) #212, 1 (1.1) #229, 1 (1.1) #3, 1 (1.1) #4, 1 (1.1) type I variant

ND, not determined.

In addition, the genotyping data of *T. gondii* were divided into several groups, including AIDS, congenital, ocular, acute toxoplasmosis, cancer and immunocompetent. Genetic characterisation of *T. gondii* isolates by PCR-RFLP based on multilocus and microsatellite markers (more than five gene markers) revealed that type II was the most predominant genotype among all groups of human toxoplasmosis (64.3%, 62.1% and 41.7% in AIDS patients, congenital form and ocular toxoplasmosis, respectively). On the other hand, the atypical type (100%) was the most common genotype in the immunocompetent group (Table 2).

Furthermore, our analysis showed that ToxoDB genotype #65 was the most prevalent in ocular (100%) and AIDS (63.6%) groups. In addition, genotypes #11 and #9 were more prevalent in the congenital (20.5%) and other groups (54.5%) (Table 3). Genetic diversity of *T. gondii* isolates in different groups of human and patients is demonstrated in Table 5.

**Table 5.** Genetic diversity of *T. gondii* isolates in different groups of patients

Category	Groups of patients	ToxoDB genotypes
All genotypes	Ocular	#65
	AIDS	#6, #9, #10, #65, #71
	Congenital	#8, #11, #36, #41, #65, #67, #108, #162, #166, # 206, #207, #208, #209, #210, #211, #212, #229
	Cancer	#9, #10, #204, #1, type I variant
Common genotype ToxoDB	All groups	#65
	Ocular and AIDS	#65
	Ocular and congenital	#65
	AIDS and congenital	#65
	AIDS and cancer	#9
Specific genotypes	Ocular	–
	AIDS	#6, #71
	Congenital	#8, #11, #36, #41, #67, #108, #162, #166, #206, #207, #208, #209, #210, #211, #212, #229
	Cancer	–

## Discussion

This systematic review is beneficial for understanding the epidemiology of *T. gondii* genotypes in high-risk groups of human. The present study determined the global prevalence rate of *T. gondii* genotypes in AIDS, congenital, ocular toxoplasmosis and other groups of patients, as well as healthy individuals. We used the documented data from the literature collected from different countries.

After searching all the databases, 26 articles were included in this study. According to the results, type II was the most predominant genotype in human toxoplasmosis during 1995–August 2017. Type I lineages are uniformly lethal with absolute lethal dose (LD_100_) of 1 in mice. On the other hand, the type II and III lineages are significantly less virulent with LD_100_⩾103. Moreover, type I or type I-like atypical isolates are more likely to cause intensive retinochoroiditis in patients and the atypical isolates often result in severe acute or disseminated toxoplasmosis in immunocompromised patients [5, 13].

Various analyses indicate that the prevalence of *T. gondii* genotypes varies in different continents. In current study, the highest and lowest prevalence of *T. gondii* type I strain were reported from American (19.6%) and African continents (6.3%). As mentioned, type I strains of *T. gondii* with LD_100_ of almost one can cause lethal infections in all species of laboratory mice even at low dose inoculations. Therefore, practicing the basic measures seems to be necessary for controlling the disease, especially in the American continent.

According to the literature, type II strains of *T. gondii* are the predominant among human samples in America, and Europe (USA, France, Portugal, etc.). Type II strains of *T. gondii* are of low virulence and high cyst-forming [5, 14, 38, 39]. We found in our analysis that Europe has the highest prevalence of *T. gondii* genotype type II (69.4%).

Similar to type II, type III strains of *T. gondii* are low virulence with LD_100_⩾10^3^ [5]. In this review, the highest prevalence of type III strains of *T. gondii* was observed in America (12/97, 12.4%). On the other hand, the lowest prevalence was reported from the European continent (12/173, 6.9%).

According to our study, in the AIDS/HIV group, genotyping of *T. gondii* was performed on 28 isolates using multilocus markers. Our analysis showed that type II strains (18/28, 64.3%) were the most common, followed by type I (6/28, 21.4%), type III (3/28, 10.7%) and mix/recombinant type (1/28, 3.6%). Toxoplasmosis in immunocompromised patients is often due to reactivation of tissue cysts usually leading to severe and life-threatening diseases, toxoplasmic encephalitis and death [40]. The predominance of type II in the patients may simply reflect the prevalence of type II infection in general population.

In order to determine the genotypes in congenital toxoplasmosis group, 214 *T. gondii* isolates were examined. Totally, 133 (62.1%), 24 (11.2%), 24 (11.2%), 17 (7.9%), 14 (6.5%) and 2 (0.9%) isolates were classified as types II, I, mix/recombinant, III, atypical and Africa I, respectively.

Congenital toxoplasmosis may result in intracerebral calcification, hydrocephalus, mental retardation and retinochoroiditis, which may be present at birth or develop later in life [41]. The role of genotypes in congenital toxoplasmosis is still controversial. However, it was shown in the study performed by Hutson *et al*. that congenital infections with type II strain are frequently associated with hydrocephalus characteristic patterns [42].

Rico-Torres *et al*. (2016) in a systematic review study demonstrated that neonatal cases infected with type II strain of *T. gondii* during the first half of pregnancy present severe clinical symptoms. The latter result suggests a crucial role for immature state and response of the foetus in susceptibility to disease [43]. Among the 26 papers included in this systematic review, only one study had determined the genotype of *T. gondii* in immunocompetent individuals and all the eight samples were infected with the atypical type.

In the ocular toxoplasmosis group, 36 isolates were genotyped and the obtained results showed that type II strains were predominant (15/36, 41.7%), followed by mix/recombinant type (9/36, 25%), type I (6/36, 16.7%), type III (4/36, 11.1%) and Africa I type (2/36, 5.6%) in this group. The previous studies in different countries revealed that ocular toxoplasmosis is associated with type I strain of *T. gondii*.

According to the studies conducted using less than five gene markers, type I strain has the ability for extracellular migration and decreases conversion to the bradyzoite form, in comparison with types II and III [44]. Our systematic review by more than five gene markers demonstrates that type II is the most prevalent in these patients. The reason for this difference can be attributed to application of more gene markers causing the genotyping to be more precise.

In recent years, researchers have used different markers (about 10–12 markers) of multilocus PCR-RFLP and microsatellites for epidemiology and genotyping studies [44]. Findings of these studies have indicated a higher genetic diversity among the different groups of patients and healthy individuals. In ToxoDB, 1457 human and animal samples were registered that revealed different ToxoDB genotypes (#1–#231). At present, major genotypes such as Chinese 1 (#9), type Br I (#6), type Br II (#11), type Br III (#8), type IV (#17) and type 12 (#4, #5) were added to the previous list (type I, II, III lineages and atypical or exotic genotypes) [38, 45].

According to new nomenclature, identified genotypes are Type I (#10), Type II (#1 clonal, #3 variant) and Type III (#2), kinds of atypical or exotic genotypes, Chinese 1 (#9, #10), Type Br I (#6), Type Br II (#11), Type Br III (#8), Type IV (#17) and Type 12 (#4, #5).

Out of the 26 studies included in the current review, 11 studies used multilocus gene markers for genotyping (ToxoDB) and determination of genetic diversity. Our analysis demonstrated that genotype #65 is predominant among all the different groups of human. Moreover, genotype #65 is the most prevalent genotype in AIDS and ocular toxoplasmosis groups. However, in the congenital toxoplasmosis group, genotype #11 was the most common. Genotypes #6 and #71 were observed only in the AIDS group. On the other hand, 16 different ToxoDB genotypes, including #8, #11, #36, #41, #67, #108, #162, #166, #206, #207, #207, #209, #210, #211, #212 and #229 were found in the congenital toxoplasmosis group. It seems that the cause of high-genetic diversity in congenital toxoplasmosis may be due to more sampling from the different regions of the world.

This review indicated that genetic diversity of *T. gondii* was low in Asian and African countries, contrary to the ones in North and South America.

## Conclusion

The current study showed that new techniques with more genetic markers for *T. gondii* genotyping, might lead to different results regarding genetic diversity. This is true especially in countries that do not have any data concerning genotyping of this parasite, such as Australia, Western and Southern Africa and Western Asia. Identification of the specific genotype in each area of the world could be valuable in developing new strategies for treatment, vaccination, diagnosis, control and prevention of *T. gondii* in human.
